# Comorbidity Between Internalising and Externalising Disorders Among Adolescents: Symptom Connectivity Features and Psychosocial Outcome

**DOI:** 10.1007/s10578-021-01264-w

**Published:** 2021-10-16

**Authors:** Cecilia A. Essau, Alejandro de la Torre-Luque

**Affiliations:** 1 University of Roehampton, London, UK; 2 Centre for Biomedical Research in Mental Health (CIBERSAM), Universidad Complutense de Madrid, Madrid, Spain; 3 Department of Psychology, Whitelands College, Roehampton University, Holybourne Avenue, London, SW15 4JD UK

**Keywords:** Comorbidity, Internalising disorders, Externalising disorders, Mental health services utilisation, Symptom network analysis, Adolescence

## Abstract

Internalising disorders are highly prevalent conditions in adolescence and tend to co-occur with externalising disorders. The present study used a symptom network approach to examine the interplay between symptoms of internalising disorders among adolescents with comorbid internalising and externalising disorders. Data comes from the National Comorbidity Survey—Adolescent Supplement, a nationally representative survey of adolescents aged 13 to 18 years. The most central symptoms across the disorders in the network were poor self-esteem and worry. The comorbidity between anxiety and depression increases the probability of having comorbid externalising disorders. Adolescents with both internalising and externalising disorders had the highest rate of health service utilisation. Comorbidity group, lifestyle factors, deficits in cognitive and academic competence and coping skills were significant covariates of the mental health outcomes. Understanding comorbidity profile of internalising and externalising disorders and central symptoms that bridge these disorders could have important clinical implications.

**Supplementary Information:**

The online version contains supplementary material available at 10.1007/s10578-021-01264-w.

## Introduction

Internalising disorders such as anxiety and depression are prevalent mental health conditions in adolescence [1]. Importantly, internalising and externalising disorders frequently co-occur [2–6]. The co-occurrence of internalising and externalising disorders is associated with greater psychosocial impairment and health service utilisation, and greater risk of relapse compared to when either disorder occurs alone [7, 8]. Reasons for the frequent comorbidity between internalising and externalising disorders remain unclear and need clarification. A common explanation is related to symptom overlap among comorbid disorders [9], however studies that examined this speculation are lacking, as they focused on examining comorbid patterns between diagnostic conditions [1]. A novel method to clarify the role of symptom overlap in the constellation of comorbid disorder is a network approach [10]. A network approach enables symptoms of multiple disorders to be combined into one network structure so that the co-occurrence of symptoms can be examined [10]. By focusing on individual symptoms and the associations between those symptoms [11] and in the way these symptoms interact with one another [12, 13] the network approach helps to identify the unique role of each individual symptom [10]. Symptoms are represented as nodes and the associations between these symptoms are represented as edges. Symptoms that connect (i.e., bridge symptoms) the different disorders increase the likelihood that an individual will develop a secondary disorder [14].

Most studies that used the network analysis approach have focused on adults with internalising disorders (i.e., anxiety and depression) [15–18]. Overall, findings of these studies showed symptoms were more connected within disorders than between disorders. In a study by Beard et al. [15], among adults with major depressive disorder (MDD) and generalised anxiety disorder (GAD), the most central symptom in this network were “sad mood” and “felt distress due to worry”. Among adult women with social phobia and MDD [18], thoughts of worthlessness was an important bridge symptom. These findings, although informative, were based on adults and may not be generalisable to adolescents. Additionally, most of these studies have important methodological shortcomings that may undermine the validity of their conclusions (e.g., small sample size, limited to female gender).

Among the few studies that have used network analysis approach among adolescents was the one by McElroy et al. [19]. This study examined the network structure of internalising and externalising disorders at three points, from middle childhood through adolescence. The most consistent disorder-level interactions were between depression and opposition defiant disorder (ODD). The most central disorders in the networks were GAD and ODD. As McElroy et al.’s study [19] was based on maternal report, the validity of the information provided might be questionable. In a recent study by de la Torre-Luque and Essau [20] among adolescents with MDD and social phobia, low self-esteem and suicidal symptoms were the most prominent in the symptom network for MDD, social phobia, and their comorbidity. This study however was limited because it focused on a limited number of comorbid disorders, i.e., MDD and social phobia. The present study expands on previous studies by examining for the first time, the network structure of the comorbidity patterns in adolescents with internalising and externalising disorders at the symptom level.

Because internalising disorders are major health issues in adolescence (particularly when co-occurring with externalising disorders), much effort has been devoted to identifying factors that place the adolescents at risk of developing these disorders. Some of the factors that have attracted research attention in recent years are those related to adolescent’s lifestyle such as physical activity, healthy diet and sleep. Studies have shown low or insufficient physical activity to be associated with internalising and externalising disorders [21, 22] and higher probability of tobacco and marijuana initiation [23, 24]. Lack of sleep has similarly been reported to be associated with internalising disorders, including anxiety and depression [25], substance abuse [26], and suicidality [27]. A recent systematic review by O’Neil et al. [28] has provided further support on the link between unhealthy dietary patterns and poor mental health. Understanding the interplay of adolescent’s lifestyle factors (i.e., physical activity, healthy diet, and sleep) within the network structure of symptoms of comorbid internalising and externalising disorders will provide useful information for the development of programmes to prevent and treat these comorbid disorders that would be more efficient and cost-effective than programmes for each specific disorder.

The main objective of the present study was to examine, using a network approach, the association between symptoms of internalising disorders in adolescents with internalising disorders and those with comorbid internalising and externalising disorders. Another aim was to examine the unique role of adolescent’s lifestyles (e.g., involvement in physical activities, sleep and eating patterns) in explaining common risk factors of comorbid internalising and externalising disorders.

## Method

### Sample

The present study used the data from the National Comorbidity Survey—Adolescent Supplement (NCS-A) [29, 30], which is a nationally representative survey of 10,123 American adolescents (48.93% boys) aged between 13 and 18 years (mean age = 15.18 years, *SD* = 1.51). Details of the NCS-A study design and survey protocols have been described in several publications [29, 31].

Data from “clinical” and “control” groups were used. The control group (CG) consisted of adolescents (*n* = 6454 [50.67% boys], 56.5% White Caucasian; mean age = 15.04 years, *sd* = 1.49) who did not meet the criteria of any mental disorders according to the Diagnostic and Statistical Manual of Mental Disorders (DSM-IV, text revised) [32], as measured using the World Health Organization Composite International Diagnostic Instrument (WMH CIDI) [33]. The clinical group comprised adolescents who met the criteria for a 12-month diagnosis of internalising (i.e., major depression, separation anxiety, social phobia, panic disorder, agoraphobia, generalised anxiety disorder) or externalising disorders (i.e., attention deficit and/or hyperactivity disorder, alcohol abuse or dependence, drug abuse or dependence, intermittent explosive disorder, conduct disorder, and/or oppositional defiant disorder).

Within the clinical group (*n* = 1781), six comorbidity patterns were formed: (1) “pure” major depression disorder (MDD group; *n* = 239 [29.29% boys], 59% White Caucasian; mean age = 15.41 years, *sd* = 1.49), (2) MDD and comorbid externalising disorders (MDD + EXT group; *n* = 174 [47.13% boys], 50.6% White Caucasian; mean age = 15.73 years, *sd* = 1.43), (3) “pure” anxiety disorders, i.e., without any externalising comorbidity (ANX group; *n* = 723 [40.94% boys], 50.9% White Caucasian; mean age = 15.15 years, *sd* = 1.50), (4) anxiety disorders and comorbid externalising disorders (ANX + EXT group; *n* = 367 [51.23% boys], 50.1% White Caucasian; mean age = 15.32 years, *sd* = 1.53), (5) internalising disorders (i.e., MDD and anxiety disorders; without comorbid externalising disorders) (ANX + MDD group; *n* = 142 [22.53% boys], 55.6% White Caucasian; mean age = 15.60 years, *sd* = 1.41), (6) internalising (anxiety and depression) and externalising disorders (ANX + MDD + EXT group; *n* = 136 [39.71% boys], 46.3% White Caucasian; mean age = 15.67 years, *sd* = 1.42).

All the participants were fluent in English and provided a written consent, signed by their parents or legal guards. None of the participants showed either comorbid eating disorders, obsessive compulsive disorder or posttraumatic stress disorder.

## Measures

Internalising and externalising disorders were diagnosed using the World Health Organization Composite International Diagnostic Instrument, version 3.0 (WMH CIDI 3.0) [33]. The WMH CIDI is a fully-structured diagnostic interview which was modified to simplify language and to use examples that are more of relevance to adolescents [31]. The interview covers a wide range of common DSM-IV disorders in adolescents (e.g., mood disorders, anxiety disorders, behaviour disorders, eating disorders, and substance use disorders). In addition to obtaining information on the 12-month diagnosis of internalising and externalising disorders, the present study focused on 15 key symptoms of internalising disorders (Table 1). Following the WMH CIDI guidelines, all diagnoses are assessed using a general screener (which comprises one or more questions related to the key symptoms of the relevant disorder), followed by specific diagnostic items for adolescents who endorsed screening questions. Only one key symptom was selected from the agoraphobia disorder module due to high rate of missing values in the other ones (>50% of participants). Concordance of WMH CIDI and DSM-IV diagnoses was endorsed in Kessler et al. [33].

**Table 1:** List of CIDI symptoms (items) included in the analyses

Domain	Label	description
Agoraphobia	ag	Fearful of being in open space
MDD	d1	Sadness
	d2	Discouraged about things in life most days
	d3	Thought about suicide
	d4	Talk/move more slowly than usual most days
	d5	Trouble concentrating most days
	d6	Low self-esteem
	d7	Felt worse than others most days
	d8	Felt guilty most days
GAD	g1	Felt distress due to worry
Panic disorder	p1	Experienced sudden attack symptoms
SAD	sa1	Being sad/uncomfortable when apart from attachment person
Social phobia	so1	Shy/afraid/uncomfortable meeting new people
	so2	Shy/afraid/uncomfortable talking to authority
	so3	Shy/afraid/uncomfortable speaking in class

*MDD * Major depressive disorder, *GAD *generalised anxiety disorder, *SAD *separation anxiety disorder

Information on the adolescent’s sociodemographic characteristics (i.e., gender, age, race, family composition and place of residence) and physical health (i.e., history of neurological, joint, respiratory, metabolic, pain-related and heart diseases; and disabilities) was collected during a face-to-face interview. Information on lifestyle patterns was also collected during the interview, including: eating patterns (regular eating pattern in terms of nutrient intake; vegetarian diet, hypo-caloric diet, other), sleep patterns (total hours of sleep during week nights and weekend nights; difficulty falling asleep which was derived from the item ‘nearly every night it took you a long time to fall asleep’; and difficulty staying asleep which was derived from item ’you woke up nearly every night and took a long time to get back to sleep’), and taking part in physical activity (frequency of light or moderate physical activity, ranging from 1 = ‘several times a week or more’ to 6 = ‘never’; this variable was derived from the item: ‘how often do you engage in light or moderate physical exercise like walking for 30 min or more?’).

The NCS-A included an 11-item scale to measure cognitive and academic competencies [31], which can be rated on a 4-point Likert scale of response (from 1 = ‘excellent’ to 4 = ‘poor’). An exploratory factor analysis (EFA) was conducted on the whole sample (*N* = 10,123) to examine factor structure underlying the scale. Principal component analysis was used to reduce dimensionality (data reduction), relying on polychoric correlation matrix. Two factors were identified, explaining 51.82% of scale variance. Both factors showed eigenvalues higher than one (factor 1 = 4.52, factor 2 = 1.18, respectively). Seven items saturated on the first factor (emotion and behaviour regulation deficits) and four items saturated on the second factor (academic/work competence deficits). The reliability indexes in the present study were satisfactory (ω between 0.74 and 0.75).

NCS-A also included a 20-item scale to measure strategies to cope with stress on a 4-point Likert scale (from 1 = ‘a lot’ to 4 = ‘not at all’) [31]. To make the results more interpretable, the response scale was recoded such that 1 = ‘not at all’ to 4 = ‘a lot’. The EFA conducted in the present study revealed a 4-factor structure, explaining 49.64% of scale variance. All these factors showed eigenvalues higher than one (from 4.07, factor 1, to 1.25, factor 4). The factors were: Factor 1 (Emotion-focused coping), Factor 2 (Problem-focused coping), Factor 3 (Cognition-focused coping), and Factor 4 (Self-focused coping). Reliability indexes were satisfactory across factors in our sample (ω between 0.61 and 0.73).

Information on physical and mental health was obtained by asking the adolescents to rate their overall physical (NCS-A item: ‘How would you rate your overall physical health?’) and mental health (NCS-A item: ‘How would you rate your overall mental health?’) on a scale which ranged from 1 (‘excellent’) to 5 (‘poor’). Information on health care service utilisation was also examined, which included: days of hospitalisation for emotional/mental problems in the past year; number of visits to mental health professionals in past year; and number of school counselling services received in the past year.

### Data Analysis

Between-group differences were examined using χ^2^-based tests, as well as Cramer’s *V* as an effect size estimate. Prediction of self-reported health (physical and mental) and mental health care service utilisation was examined using generalised linear modelling (GLM). Study groups, lifestyle patterns, coping strategies, as well as cognitive and academic competencies were considered as covariates. Physical and mental health outcomes were modelled under gamma distribution; health service utilisation outcomes were modelled under negative binomial distribution, as high proportion of adolescents was expected to have no mental health service use. A lower Akaike information criterion (AIC) was used to inform fit of the model with covariates in comparison to the model without them. Model comparison relied on a stepwise approach: a model without covariates (unconstrained model), a model with a study group as a covariate, and the model with all covariates. Odds ratio (*OR*) was used to report parameter loadings. A significant difference from one loading was detected by means of Wald’s test under a *t*-based distribution.

A network analysis (NA) approach [34] was used to examine the relations between internalising symptoms across the six study groups. This approach focuses on the complex patterns of relations between symptoms that underlie a specific mental health condition as well as the relations of these symptoms with symptoms of various other mental health conditions. In the graphical representation of NA, the nodes represent the symptoms and edges between them reflect their conditional dependence/relation (i.e., association between two symptoms after controlling for all other associations between the symptoms in the network). The nodes with stronger correlations are placed near the centre and show their influencing (central) role in keeping the disorders stable [35]. Network was weighted and regularised (under regularised logistic regression framework) by shrinking small connections in the network (set to be exactly zero) due to Holm’s correction for multiple comparison testing. Nested Lasso regressions were used for network estimation, with model selection based on the extended Bayesian information criterion (EBIC) and penalisation based on a gamma hyperparameter (γ = 0.25).

Data of participants in the clinical groups were used for the NA. Those with a high rate of missing values (i.e., ten or more symptoms without response) were excluded. Multiple imputation procedures were used to estimate missing values, with a cut-off point of 10 multiple imputations and 50 iterations to obtain convergence for the solution comprising the imputed values [36]. Estimation method relied on the random forest algorithm. The algorithm is suitable to handle data violating normality assumptions and highly recommended for high dimensionality data (i.e., high correlations between items) [37, 38].

The network comparison test was used to examine the similarity of networks across the study groups [39, 40]. Specifically, the test investigates network invariance at three levels: network structure (i.e., whether the structure of both networks is invariant between groups), global strength (i.e., invariant overall connectivity of symptoms across between groups) and edge strength (i.e., whether each association between symptoms is invariant across groups, using a Bonferroni-Holm correction to prevent from multiple testing bias). Edge strength invariance was tested when the network structure showed no invariant between groups. Two sets of pairwise network comparisons were carried out: First, we compared groups with internalising disorders (first set: MDD vs. ANX, MDD vs. MDD + ANX, ANX vs. MDD + ANX; second set: MDD + EXT vs. ANX + EXT, MDD + EXT vs. MDD + ANX + EXT, ANX + EXT vs. MDD + ANX + EXT). Second, we compared networks of groups with versus without comorbid externalising disorders (MDD vs. MDD + EXT, ANX vs. ANX + EXT, ANX + MDD vs. ANX + MDD + EXT groups). A Bonferroni-based correction on *p* level was applied to prevent from multiple comparison testing (0.05/6 = 0.0083, for externalising-comorbidity network comparison; and 0.05/3 = 0.0166, for internalising-disorder subtype comparison).

Centrality measures were calculated to examine the role of each symptom within the network across the six groups [35]. Two centrality measures were calculated: strength (i.e., sum of the edge weights connected to a node) and betweenness (i.e., number of times that node lies on the shortest path between two other nodes). Network clustering were measured using the following indicators [41–43]: transitivity (i.e., how two nodes which share a neighbour are interconnected within the network; global clustering property of the entire network), the average of shortest paths between nodes, and small worldness index (property to have both a high clustering coefficient and a short average path length; values higher than 1 indicate that the network has the small-world property). Network robustness tests were conducted under non-parametric bootstrapping [44].

Finally, to investigate the association between network properties and self-reported health/and mental health service utilisation, non-parametric correlation analysis (Spearman’s correlation) was conducted between NA properties (network global strength, average of weighted correlations and clustering properties) and the outcome measures (i.e., self-reported physical and mental health, and health care service utilisation).

All the analyses were conducted using R Core Software [45], packages mice, lme4, qgraph, igraph, bootnet and NetworkComparisonTest.

## Results

Minor differences were found across the study groups (Table 2). Within the anxiety disorders, the most prevalent disorder was social phobia. The ANX + MDD, compared to the pure anxiety group, showed higher proportion of GAD and agoraphobia, although social phobia was also common. The most prevalent externalising disorders was intermittent explosive disorder with more than 50% of adolescents having this disorder across the externalising comorbid groups. Alcohol and drug abuse disorders were more prevalent in the MDD + EXT group, whereas oppositional defiant disorder was more prevalent in the ANX + MDD + EXT group (Table 2).

**Table 2:** Sociodemographic characteristics of the study groups

	Study groups							Contrast test	Effect size
	CG	MDD	MDD + EXT	ANX	ANX + EXT	ANX + MDD	ANX + MDD + EXT		
*N*	6454	239	174	723	367	142	136		
Gender (% girls)	49.33	70.71	52.87	59.06	48.77	77.47	60.29	107.75	0.11
Age								97.74	0.07
Early (13–14 years)	41.85	30.96	20.69	39.00	34.88	26.06	22.79		
Mid (15–16 years)	38.05	40.17	44.83	38.73	38.96	42.25	41.91		
Late (17–18 years)	20.10	28.87	34.48	22.27	26.16	31.69	35.29		
Race								46.23	0.04
White	56.49	59.00	50.57	50.90	50.14	55.63	46.32		
Hispanic	18.44	15.90	29.89	19.23	22.34	18.31	25.74		
Black	19.17	18.41	10.92	21.72	19.89	17.61	21.32		
Other	5.90	6.69	8.62	8.16	7.63	8.45	6.62		
Parents’ education								72.46	0.05
< High school	15.74	12.97	18.97	18.53	22.34	15.49	19.12		
High school graduate	30.23	23.43	35.06	35.27	31.34	30.28	34.56		
Some college	18.48	25.52	20.69	17.98	23.43	16.20	22.06		
College graduate	35.54	38.08	25.29	28.22	22.89	38.03	24.26		
Household income^†^								25.84	0.04
Low	16.35	12.97	19.54	19.64	17.17	18.31	22.06		
Low-average	19.93	19.67	20.11	19.09	23.16	14.79	23.53		
High-average	30.65	31.38	25.29	31.95	30.52	35.21	30.15		
High	33.08	35.98	35.06	29.32	29.16	31.69	24.26		
Urbanicity								28.96	0.04
Census major metropolitan area	43.59	44.77	48.85	44.26	45.23	53.52	44.12		
Other urbanised county	31.84	33.47	29.89	36.65	36.51	26.76	36.03		
Rural county	24.57	21.76	21.26	19.09	18.26	19.72	19.85		
Biological parents living with adolescent								114.95	0.08
No parents	7.79	10.88	17.82	10.24	10.35	11.97	13.97		
One parents	34.58	38.91	40.23	41.63	50.14	43.66	45.59		
Both parents	57.62	50.21	41.95	48.13	39.51	44.37	40.44		
Physical diseases* (%)	51.75	66.11	77.01	62.79	72.21	73.24	77.94	182.97	0.14
Anxiety disorders									
Agoraphobia				8.58	13.35	18.31	20.59	23.41	0.07
Generalised anxiety				5.39	6.81	21.13	18.38	55.26	0.10
Panic disorder				7.61	13.35	13.38	17.65	17.80	0.06
Separation anxiety				5.95	8.99	7.75	14.71	13.16	0.05
Social phobia				80.77	77.93	72.54	75.00	6.27	0.03
Externalising disorders									
ADHD			14.94		13.07		19.12	2.88	0.04
Alcohol abuse			28.16		18.53		16.91	8.15	0.07
Alcohol dependence			4.60		3.00		5.15	1.60	0.03
Conduct disorder			20.69		14.44		19.12	3.82	0.04
Drug abuse			32.18		21.80		25.00	6.77	0.06
Drug dependence			10.34		5.18		7.36	4.92	0.05
Intermittent explosive disorder			54.60		59.95		52.21	2.98	0.04
Oppositional defiant disorder			21.26		25.34		36.76	10.03	0.07

Percentage of participants is displayed by study groups. χ^2^ tests for between-category differences and related effect size estimates (Cramer’s *V*) are presented

*CG *Control group, *MDD *depression group, *MDD + EXT *depression with comorbid externalising disorder group, *ANX *anxiety group, *ANX + EXT *anxiety with comorbid externalising disorder group, *ANX + MDD *comorbid anxiety and depression group, *ANX + MDD + EXT *comorbid depression and anxiety with comorbid externalising disorder group, *ADHD *attention deficit hyperactivity disorder

^†^Levels based on poverty line

*Physical diseases included in the analyses were: joint problems (arthritis, rheumatism, chronic back/neck problems), neurological diseases (migraine, epilepsy), respiratory problems (seasonal allergies, asthma), heart problems, chronic pain and metabolic diseases (stomach/intestine ulcers, diabetes)

Table 3 summarises model fit indexes and estimates of outcome prediction models. The descriptive statistics of the outcomes by group are displayed in the Supplementary Table S1. Model with all the covariates showed a better fit to data than the unconstrained and group-covariate models, suggesting a significant contribution of the covariates (i.e., study group, lifestyle factors, competence deficits, coping skills) to the study outcomes, except for the case of hospital admissions; the model with all the covariates did not converge and the model with the comorbidity group fitted better to data than the unconstrained one. However, estimates were quite inaccurate, as observed by large confidence interval of coefficients. The six study groups (in comparison to the control group) had poorer health and used more mental health services (i.e., mental health care visits and school counselling services). The group with comorbid internalising and externalising disorders (ANX + MDD + EXT) had the highest rate of mental health services utilisation. MDD and the ANX + MDD groups also showed higher *odds ratio* than the anxiety groups (ANX) to have used mental health services.

**Table 3:** Outcome prediction models and covariate loadings

	Mental health		Physical health		Hospital admission		Mental health care visits		School Counselling services	
	*OR* (*CI*_*95*_)	*t* value	*OR* (*CI*_*95*_)	*t* value	*OR* (*CI*_*95*_)	*Z* value	*OR* (*CI*_*95*_)	*Z* value	*OR* (*CI*_*95*_)	*Z* value
Study group (ref. = CG)										
MDD	0.94 (0.92, 0.96)	− 6.34**	0.97 (0.95, 0.99)	− 3.56**	3.84 (0.48, 513.79)	0.92	1.76 (1.57, 1.98)	9.36**	1.16 (1.02, 1.31)	2.35*
MDD + EXT	0.94 (0.92, 0.96)	− 5.67**	0.98 (0.96, 1.00)	− 2.00*	2.03 (0.19, 1164.58)	0.41	2.05 (1.79, 2.35)	10.39**	1.30 (1.13, 1.49)	3.80**
ANX	0.96 (0.95, 0.98)	− 5.29**	0.98 (0.97, 0.99)	− 3.03**	0.93 (0.21, 8.81)	− 0.08	1.11 (1.03, 1.21)	2.54*	1.07 (0.99, 1.16)	1.84
ANX + EXT	0.96 (0.95, 0.98)	− 4.54**	0.98 (0.97, 1.00)	− 2.34*	6.44 (1.08, 219.39)	1.57	1.83 (1.66, 2.02)	11.95**	1.44 (1.31, 1.58)	7.53**
ANX + MDD	0.95 (0.93, 0.98)	− 3.86**	0.97 (0.95, 0.99)	− 2.54*	7.20 (0.64, 135.42)	1.05	2.05 (1.77, 2.39)	9.42**	1.17 (0.99, 1.36)	1.93
ANX + MDD + EXT	0.96 (0.93, 0.98)	− 3.68**	0.98 (0.96, 1.00)	− 1.41	9.07 (0.78, 227.43)	1.15	2.73 (2.36, 3.16)	13.54**	1.62 (1.40, 1.87)	6.62**
Physical diseases (ref. = no disease)	1.00 (0.99, 1.00)	− 1.05	0.99 (0.97, 0.99)	− 4.18**			1.02 (0.98, 1.07)	0.95	1.06 (1.01, 1.10)	2.34*
Eating patterns (ref. = no diet)										
Vegetarian	1.03 (0.98, 1.03)	0.21	0.97 (0.95, 0.99)	− 2.96**			1.10 (0.95, 1.27)	1.29	1.10 (0.96, 1.26)	1.33
Hypo-caloric	0.99 (0.97, 1.02)	− 0.58	1.00 (0.98, 1.02)	− 0.33			0.94 (0.82, 1.07)	− 0.95	0.94 (0.82, 1.12)	− 1.07
Others	1.00 (0.99, 1.00)	− 0.26	0.97 (0.95, 0.99)	− 2.45*			1.11 (0.93, 1.33)	1.18	0.94 (0.78, 1.12)	− 0.70
Physical activity	0.99 (0.99, 1.00)	− 5.24**	0.98 (0.98, 0.99)	− 15.14**			0.99 (0.98, 1.01)	− 0.85	0.98 (0.97, 0.99)	− 2.36*
Sleep patterns										
Sleep time^1^	1.00 (1.00, 1.00)	1.49	1.00 (1.00, 1.01)	3.15**			1.03 (1.01, 1.05)	3.13**	1.03 (1.01, 1.05)	3.41**
Difficulty falling asleep (ref. = no)	0.99 (0.98, 1.00)	− 1.87	0.99 (0.98, 1.00)	− 1.28			1.19 (1.12, 1.27)	5.52**	0.98 (0.92, 1.04)	− 0.76
Difficulty staying asleep (ref. = no)	0.98 (0.96, 0.99)	− 3.70**	0.99 (0.98, 1.00)	− 1.61			1.17 (1.09, 1.26)	4.09**	1.16 (1.07, 1.24)	3.89**
Deficits in individual competencies										
Emotion and behaviour regulation	0.99 (0.98, 0.99)	− 18.96**	0.99 (0.99, 0.99)	− 10.02**			0.99 (0.99, 1.00)	− 1.49	1.01 (1.01, 1.02)	3.44**
Academic/work competencies	0.99 (0.99, 1.00)	− 6.22**	0.99 (0.99, 0.99)	− 8.86**			1.03 (1.02, 1.05)	5.32**	1.01 (0.99, 1.02)	0.87
Coping skills										
Emotion-focused	0.99 (0.99, 1.00)	− 8.45**	1.00 (1.00, 1.00)	− 4.05**			1.00 (1.00, 1.01)	1.22	1.01 (1.01, 1.02)	3.70**
Problem-focused	1.01 (1.01, 1.01)	7.95**	1.00 (1.00, 1.00)	1.81			0.99 (0.98, 1.00)	− 2.55*	0.99 (0.98, 1.00)	− 2.92**
Cognition-focused	1.00 (1.00, 1.00)	− 1.68	1.00 (1.00, 1.01)	0.69			1.00 (0.99, 1.01)	− 0.12	1.00 (1.00, 1.01)	1.37
Self-focused	1.00 (1.00, 1.01)	2.23*	1.00 (1.00, 1.00)	0.77			0.99 (0.98, 1.01)	− 0.93	0.99 (0.97, 1.01)	− 1.33
(Intercept)	1.98 (1.90, 2.06)	33.78**	1.79 (1.72, 1.86)	30.24**	0.03 (0.02, 0.05)	− 12.58**	0.94 (0.72, 1.22)	− 0.49	0.69 (0.54, 0.88)	− 2.93**
AIC (model all covariates)	19,299.19		21,228.57		1200.23		31,370.32		26,078.47	
AIC (group covariate)	20,431.08		22,029.39		894.44		25,911.22		21,859.50	
AIC (unconstrained model)	24,068.05		25,445.30		ns		25,492.42		21,662.58	

Estimates from generalised linear model were calculated considering gamma outcome distribution for either self-reported mental and physical illness; and Poisson distribution for health services utilisation (i.e., hospital admission, mental health services utilisation, and school counselling services). Three regression models were compared for each outcome: the unconstrained one (model without covariates), the group-covariate model (including the study group as a covariate) and a model with all the covariates. Estimates displayed relied on the all-covariate models

*ref.* Reference category (category that is compared with the category of interest on this factor); *OR* odds ratio; *CI*_95_ 95% confidence interval; *t value* Statistic derived from the *t*-based test, *AIC* Akaike information criterion, *CG* control group, *MDD* depression group, *MDD + EXT* depression with comorbid externalising disorder group, *ANX* anxiety group, *ANX + EXT* anxiety with comorbid externalising disorder group, *ANX + MDD* comorbid depression and anxiety group, *ANX + MDD + EXT* comorbid depression and anxiety with comorbid externalising disorder group

^1^Number of hours individual sleep

*p < 0.05; **p < 0.01

For lifestyle factors, doing light physical activities was associated with better physical and mental health, and higher uses of school counselling services. Sleep patterns (especially “difficulty staying asleep”) were related to worse self-reported mental health. Higher scores in deficits cognitive competencies were related to poorer self-reported health. Moreover, deficits in emotion and behaviour regulation were related to lower visits to mental health professionals, but higher school counselling services; deficits in academic competencies score were associated with higher visits to mental health professionals. On coping skills, the use of emotion-focused strategies was a significant covariate of poor mental health and high use of mental health services and school counselling services. The opposite was found for the use of problem-focused coping strategies.

### Network Estimation and Association with Outcomes

After removing the participants with 10 or more missing symptoms (Table 1), data of 1173 participants (65.86% of initial clinical sample) were used for the NA. This sample did not differ significantly from the initial clinical group on their sociodemographic and health-related features (Supplementary material Table S2). A total of 5464 missing values were estimated by multiple imputations, which made up of 31.05% of the data. Distribution of data with imputed values showed a similar data distribution as the original data (Supplementary material Figure S1).

**Fig. 1:**
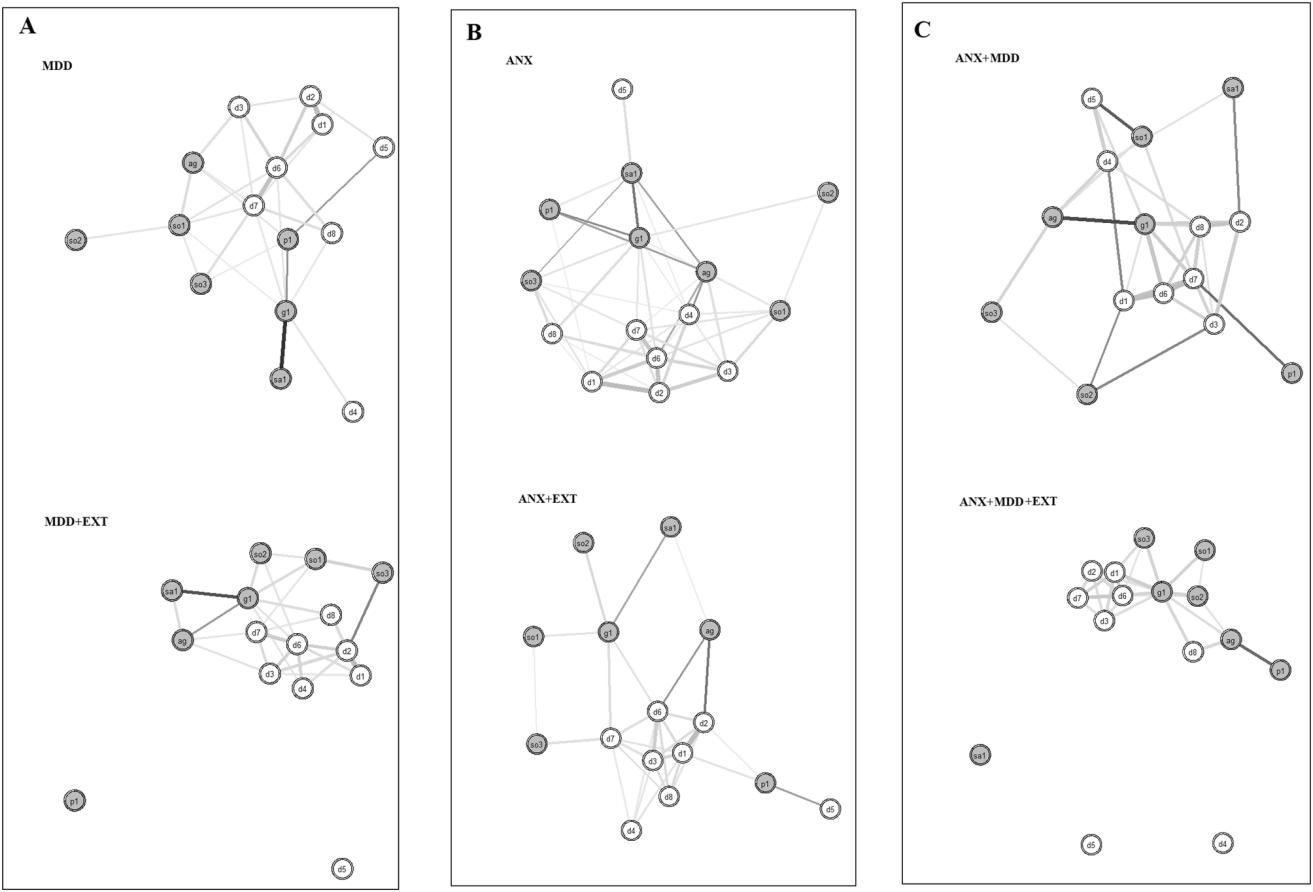
Estimated symptom constellations across the study groups. *Box A* Major depression group networks, *Box B* Anxiety group networks, *Box C* comorbid depression and anxiety groups networks, *MDD* depression group, *MDD + EXT* depression with comorbid externalising disorder group, *ANX* anxiety group, *ANX + EXT* anxiety with comorbid externalising disorder group, *ANX + DEP* comorbid depression and anxiety group, *ANX + DEP + EXT* comorbid depression and anxiety with comorbid externalising disorder group. Edges represent connections between symptoms (positive partial correlations in grey or negative correlations in black). The thicker the edge, the stronger the connection. Nodes in grey depict anxiety symptoms. Nodes in white depict major depression symptoms. The colouring of the white circle around each node represents the explained variance by its neighbours. Symptoms: ag = Fearful of being in open space. d1 = Sadness. d2 = Discouraged about things in life most days. d3 = Thought about suicide. d4 = Speak/move more slowly than usual most days. d5 = More trouble concentrating most days. d6 = Low self-esteem. d7 = Felt worse than others most days. d8 = Felt guilty most days. g1 = Felt distress due to worry. p1 = Experience sudden attack. sa1 = Being sad/uncomfortable when apart from attachment person. so1 = Shy/afraid/uncomfortable meeting new people. so2 = Shy/afraid/uncomfortable talking to authority. so3 = Shy/afraid/uncomfortable speaking in class

The estimated networks for the study groups are depicted in Fig. 1. The resulting graphs were estimated after forcing low between-symptom correlations to exactly zero, based on Holm’s correction. All the symptoms were present in the network constellations across the study groups, except for the MDD + EXT and ANX + MDD + EXT groups. The symptom related to concentration problems (d5) showed no significant connections with other symptoms in the network of both these groups (i.e., MDD + EXT and ANX + MDD + EXT); other symptoms also appeared to be unconnected in these two groups (i.e., sa1, d4 and p1). The correlation matrices across the study groups are displayed in Tables S3A through S3F (Supplementary material).

**Fig. 2:**
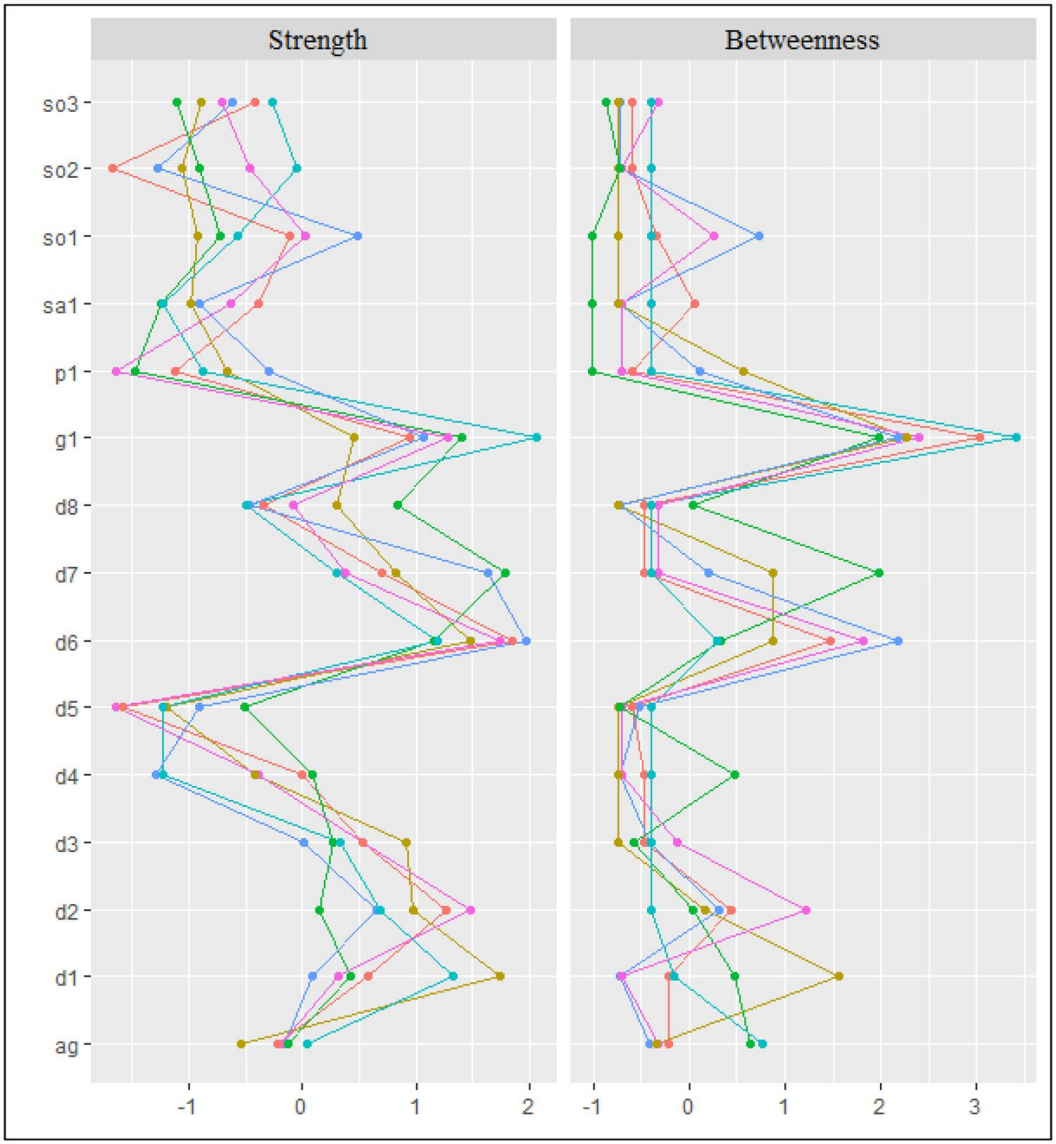
Centrality measures across study groups. Orange line = Anxiety group network. Olive green line = Anxiety + externalising comorbidity group network. Green line = Comorbid depression and anxiety group network. Turquoise line = Comorbid depression and anxiety + externalising comorbidity group network. Measures are displayed on a relative scale from 0 (lowest) to 1 (highest). Blue line = Depression group network. Pink line = Depression + externalising comorbidity group network. ag = Fearful being in open space. d1 = Sadness. d2 = Discouraged about things in life most days. d3 = Thought about suicide. d4 = Speak/move more slowly than usual most days. d5 = More trouble concentrating most days. d6 = Low self-esteem. d7 = Felt worse than others most days. d8 = Felt guilty most days. g1 = Felt distress due to worry. p1 = Experience sudden attack symptoms. sa1 = Being sad/uncomfortable when apart from attachment person. so1 = Shy/afraid/uncomfortable meeting new people. so2 = Shy/afraid/uncomfortable talking to authority. so3 = Shy/afraid/uncomfortable speaking in class

The centrality measures for the network constellations are displayed in Fig. 2. The most influential (central) symptoms across the networks were the depression-related symptom ‘low self-esteem’ (d6) and the anxiety-related symptom related to ‘emotional distress experience due to worry’ (g1). Those symptoms showed higher levels of both strength and betweenness measures (except in the case of the groups with comorbid ANX + MDD which showed attenuated betweenness). Other central symptoms for the internalising comorbidity groups were: ‘speak/move more slowly than usual most days’ (d4) and ‘felt worse than other most days’ (d7) for the ANX + MDD group (i.e., higher levels in both centrality measures); and ‘sadness’ (d1) and ‘shy/afraid/uncomfortable when meeting new people’ (so1) for the ANX + MDD + EXT group (with higher levels of both centrality measures) and the ANX + MDD + EXT group (with higher levels of strength). Furthermore, higher-than-1 small worldness indexes were found across networks, but not in the MDD + EXT and the ANX + MDD groups (index higher than 0.90). The networks with higher clustering measures were in the ANX + EXT group, followed by the ANX + MDD + EXT group (Supplementary Material Table S4).

The network comparison based on the type of disorder (i.e., internalising or externalising disorder) showed similar network structure across the study groups. However, differences were found between the MDD and ANX + MDD groups in terms of the global strength, with higher overall edge strength found in the MDD network (*A*_*MDD*_ = 8.24) in comparison to the ANX + MDD network (*A*_*ANX + MDD*_ = 1.00), with absolute difference in edge strength = 7.24, *p* = 0.008. Further analyses showed no significant differences between networks of individuals with externalising and internalising disorders (i.e., MDD vs. MDD + EXT, ANX vs. ANX + EXT, ANX + MDD vs. ANX + MDD + EXT groups) nor the networks of individuals with internalising and externalising comorbid conditions (i.e., MDD + EXT vs. ANX + EXT, MDD + EXT vs. MDD + ANX + EXT, ANX + EXT vs. MDD + ANX + EXT).

Bootstrapped tests to study network robustness are reported in the Supplementary Fig. S2. Network estimates (edges) matched with those from the bootstrapped samples, thus providing evidence on network robustness.

**Fig. 3:**
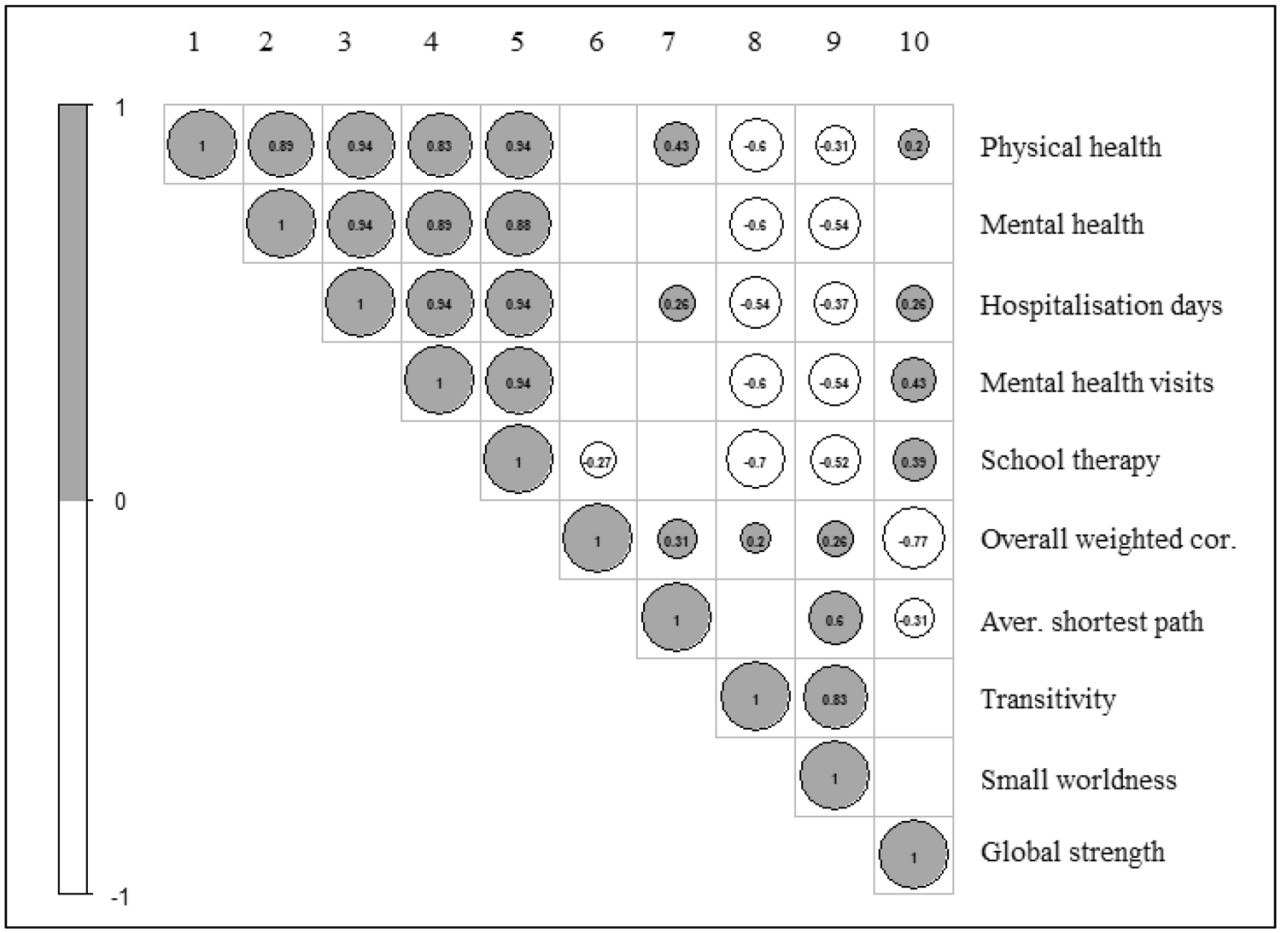
Correlation plot of symptom network properties and outcomes. Spearman’s rank correlation coefficients were estimated. Correlations equal or higher than 0.20 were displayed. All correlations were significant with *p* < 0.01. Study outcomes (based on average scores across study groups): physical health; mental health; hospitalization days; mental health professional visits; and school counselling/therapy sessions. Symptom-network properties: overall weighted correlations, clustering measures (average shortest path, transitivity and small worldness index), global edge strength. *cor.* correlations, *Aver.* average

In the final analyses, we examined the associations between the NA properties and the overall outcomes across the clinical groups. As shown in Fig. 3, global edge strength correlated positively with all the outcomes except with the self-reported mental health. Transitivity and small worldness index correlated negatively with all the outcomes. These findings suggested that the lower indexes were associated with poorer self-reported health and higher mental health services utilisation.

## Discussion

To our knowledge, this is the first study that have examined the complex patterns of network relations of internalising symptoms among adolescents with internalising disorders with or without comorbid externalising disorders using a symptom network approach. This is also the first study that has investigated the health outcomes (i.e., self-rated physical and mental health, and mental health service utilisation) and lifestyles which were used to characterise specific comorbidity patterns.

In line with previous studies [2–4], anxiety and MDD co-occur frequently, with more than 15% of adolescents showing more than one type of internalising disorders. Additionally, 41.5% of adolescents with anxiety and/or MDD showed comorbid externalising disorders. The explanation of this finding is not completely clear. As argued by McElroy et al. [19], the co-occurrence of disorders may be due to the direct influence of each other. They gave an example of the comorbidity between anxiety and depression,when anxiety normally begins before depression; in such cases, anxiety is often regarded as placing an individual at risk for developing depression [46]. Other authors argued that the co-occurrence between anxiety and depression might be related to cognitive/neurophysiological processes that led to an exhaustion of the body which is manifested as depression [47]. McElroy et al. [19] also speculated that externalising behaviour may indirectly lead to internalising problems through mediating variables [48, 49]. Specifically, disruptive behaviours may lead to negative reactions from parents and other significant others, which in turn may foster feelings of irritability, distress and worthlessness within the adolescents [49]. Further studies are needed to investigate these speculations.

Adolescents with internalising disorders showed poorer physical and mental health and higher use of services compared to those without any disorders. Our findings also showed that individuals with MDD (with or without comorbid anxiety) showed higher service utilisation, whereas those with comorbid externalising disorders showed the highest amount of service utilisation. This finding replicated the previous study by Essau [7] that reported adolescents with anxiety and comorbid disorders tended to be more psychologically distressed and used more mental health services than adolescents with anxiety disorders only. Psychological distress and mental health services utilisation increased with the number of comorbid disorders; that is, the more comorbid disorders the adolescents have, the more distressed they were and the more likely they were to have sought professional help. Lewinsohn et al. [6] similarly found the presence of comorbid disorders to be related to higher use of mental health services. Specifically, adolescents with anxiety disorders with comorbid substance use disorders, or with comorbid disruptive behaviours had higher rates of mental health services utilisation compared to those with anxiety disorders only.

In terms of network properties, clustering measures may be a potential marker of comorbidity pattern severity in adolescents with internalising disorders. We found that clustering measures (i.e., transitivity and small worldness index) correlated negatively with self-rated health (i.e., the higher the measures the poorer the self-rated health) and service utilisation (the higher the measures the lower the service use). Network global strength was also associated with those health-related outcomes. However, our results should be interpreted with caution because we used a non-parametric analysis and that the number of observations in analysis was low. Clustering and global strength properties help provide a global picture on the complexity of the pattern of relations between symptoms within the network [36, 37]. Some studies have provided mixed evidence on the usefulness of complexity-related measures to study mental disorder severity and prognosis [50, 51]. For instance, Beard et al. [15] found increased connectivity in the network over the course of treatment, although symptom severity decreased. Conversely, Van Borkulo et al. [52] showed that symptom network of patients with persistent MDD at follow-up was more densely connected than the one from patients who recovered from the disorder.

Lifestyle factors such as sleep routines and involvement in physical activity seemed to have an important role in determining adolescent’s health outcome and the use of health/mental health services. Indeed, low involvement in physical activity and lack of sleep have been reported to be associated with internalising and externalising disorders [21, 22, 25]. Sleep problems, related to insufficient sleep time, may aggravate both externalising and internalising disorders, through mood disturbances, attention problems or aggressive behaviour [53, 54]. For physical activity, two potential mechanisms have been proposed to explain its protective effects: its involvement in preventing physical problems (e.g., obesity and metabolic dysregulation) and its contribution to self-control and efficient emotion regulation [55, 56].

Influential items across study groups were low self-esteem (MDD symptoms) and worry (anxiety symptoms). These symptoms might have a bridging role because they were central across disorders, as higher centrality values in both measures (i.e., strength and betweenness) were observed across the study groups. First, symptoms with higher strength (roughly understood as the sum of all the correlations with other symptoms) may spread activation more widely. This makes these symptoms as targets for monitorisation (i.e., changes in the disorder status may be manifested in these symptoms earlier) and treatment (i.e., central symptom amelioration may activate symptom reductions in the whole network of constellation). On the other hand, symptoms with higher betweenness values may be likely to spread to several syndromic clusters (disorders), leading to a comorbid presentation, subsequently. A recent study by de la Torre-Luque and Essau [20] which focused on adolescents with MDD and social phobia, similarly shown low self-esteem as being central in the symptom network for pure as well as for comorbid disorders. This finding supports the importance of self-esteem in determining emotional well-being [57]. As shown in several studies, low self-esteem is associated with a wide range of mental disorders, such as anxiety, depression, suicidal tendencies, and eating disorders [58]. Indeed, a meta-analysis [59] has documented low self-esteem as an important risk factor for the development of both MDD and anxiety disorders in adolescence.

In the comorbid internalising group, as well as in the internalising and externalising group, other key symptoms were mostly related to MDD (i.e., ‘sadness’ in the ANX + MDD + EXT group with higher strength values; ‘speak/move more slowly’ and ‘felt worse than others most days’ in the ANX + MDD group with higher strength and betweenness values) and social phobia (i.e., ‘shy/afraid/uncomfortable when meeting new people’ in the ANX + MDD + EXT group with higher strength values). Social phobia symptoms may be highly connected with poor social competence and poor behavioural adjustment. For that reason, we speculate that these symptoms may contribute to depression development (through varying paths, for instance, the relation with thoughts of worthlessness in social situations) and may have a mediation role in the development of externalising symptoms. As reported in some studies, low social competence and emotion regulation problems in social contexts may lead to the development of both externalising and internalising disorders later in life [60, 61]. McElroy et al. [19] showed the emergence of two regions of clustered nodes which reflected a strong link between internalising and externalising disorders, which were bridged via the edges generalised anxiety disorder-attention deficit hyperactivity disorder (GAD-ADHD), and depression-oppositional defiant disorder (DEP-ODD). As the study by McElroy et al. [19] focused on diagnoses, it is not clear from that research, which symptoms played a central role in bridging the link between internalising and externalising disorders.

The present study is not without limitations. First, the information about psychiatric symptoms was based on cross-sectional assessment which makes it impossible to draw any conclusions about the temporal relation between symptoms. Second, the analyses were focused on diagnosable disorders to identify patterns of symptom relationships between internalising and externalising disorders. A more comprehensive design would be to focus on participants with subclinical syndromes and to follow them up to adulthood; such design would enable tracking the development of disorders from early stage to diagnosable conditions. Finally, there was a large number of imputed missing data. However, it is also important to note that distribution of imputed data preserved the original data distribution. These limitations notwithstanding, this study was the first to have examined the associations between symptoms of internalising and externalising disorders among adolescents with pure internalising (anxiety and MDD) and their comorbidity with externalising disorders.

Findings of the present study could have clinical implications in designing prevention protocols for adolescents with internalising disorders. First, lifestyle management that includes sleep hygiene and involvement in physical activity should be considered when developing a prevention programme. Second, our findings emphasise the importance of considering a transdiagnostic programme for the prevention of internalising disorders in adolescents given the centrality of common symptoms across disorders. Specifically, low self-esteem and worry are key symptoms that bridge internalising and externalising disorders. Another key bridge symptom is “being shy or feeling uncomfortable when meeting new people”, which could be an indicative of low social skills.

## Summary

The present study used a symptom network approach to examine the comorbidity between symptoms of internalising and externalising disorders and their complex associations. It also explored health outcomes (i.e., self-rated physical and mental health, and mental health service utilisation) and lifestyles (i.e., involvement in physical activities, sleep pattern, type of food consumed) which might be related to specific comorbid patterns. The study used the data from the National Comorbidity Survey—Adolescent Supplement (NCS-A), which is a nationally representative survey of 10,123 American adolescents (48.93% boys) aged between 13 and 18 years (mean age = 15.18 years, SD = 1.51). Data from “clinical” and “control” groups were used. The control group consisted of adolescents (n = 6454) who did not meet the criteria of any mental disorders. The clinical group comprised adolescents who met the criteria for a 12-month diagnosis of internalising (i.e., major depression, separation anxiety, social phobia, panic disorder, agoraphobia, generalised anxiety disorder) or externalising disorders (i.e., attention deficit and/or hyperactivity disorder, alcohol abuse or dependence, drug abuse or dependence, intermittent explosive disorder, conduct disorder, and/or oppositional defiant disorder). In the clinical group, six comorbidity patterns were identified: “pure” major depressive disorder (MDD), MDD and externalising disorders, “pure” anxiety disorders, anxiety and externalising disorders, internalising disorders (depression and anxiety disorders), and internalising and externalising disorders. Result showed that the most central symptoms across the disorders in the network were poor self-esteem and worry. The comorbidity between anxiety and depression increases the probability of having comorbid externalising disorders. Adolescents with both internalising and externalising disorders had the highest rate of health service utilisation. Comorbidity group, lifestyle factors, deficits in cognitive and academic competence and coping skills were significant covariates of the mental health outcomes. Understanding comorbidity profile of internalising and externalising disorders and central symptoms that bridge these disorders could have important clinical implications.

## Supplementary Information

Below is the link to the electronic supplementary material.

Supplementary material 1 (DOCX 441.9 kb)
