# Supplementary material for: Comorbidity Between Internalising and Externalising Disorders Among Adolescents: Symptom Connectivity Features and Psychosocial Outcome
Source: Child Psychiatry Hum Dev. 2021 Oct 16;54(2):493–507. doi: 10.1007/s10578-021-01264-w (PMC9977855; doi:10.1007/s10578-021-01264-w)
Supplement: Supplementary file 1 — Supplementary material 1 (DOCX 441.9 kb) [file 10578_2021_1264_MOESM1_ESM.docx]

## **Supplementary material**

| *Table S1.* Descriptive statistics of outcomes. | | | | | | |  |
| --- | --- | --- | --- | --- | --- | --- | --- |
|  | Study groups | | |  |  |  |  |
|  | CG | MDD | MDD+EXT | ANX | ANX+EXT | ANX+MDD | ANX+MDD+EXT |
| Mental health |  |  |  |  |  |  |  |
| Excellent | 31.66 | 11.34 | 10.40 | 20.06 | 13.11 | 13.38 | 6.67 |
| Very good | 40.24 | 36.97 | 26.59 | 34.40 | 30.60 | 29.58 | 25.93 |
| Good | 24.36 | 39.92 | 37.57 | 36.07 | 40.16 | 40.85 | 41.48 |
| Fair | 3.53 | 10.50 | 23.12 | 9.05 | 13.66 | 14.79 | 20.00 |
| Poor | 0.20 | 1.26 | 2.31 | 0.42 | 2.46 | 1.41 | 5.93 |
| Physical health |  |  |  |  |  |  |  |
| Excellent | 18.71 | 8.37 | 9.20 | 13.16 | 12.26 | 6.34 | 8.82 |
| Very good | 38.75 | 31.80 | 31.61 | 32.41 | 28.61 | 32.39 | 24.26 |
| Good | 32.57 | 44.35 | 32.18 | 38.64 | 36.24 | 42.25 | 38.97 |
| Fair | 9.25 | 13.81 | 25.86 | 14.13 | 18.80 | 16.20 | 25.74 |
| Poor | 0.71 | 1.67 | 1.15 | 1.66 | 4.09 | 2.82 | 2.21 |
| Hospital admissions (in days) | 0.03 (0.66) | 0.11 (0.77) | 0.06 (0.47) | 0.03 (0.24) | 0.18 (3.14) | 0.20 (1.66) | 0.26 (1.75) |
| Mental health care visits | 0.27 (2.40) | 1.53 (6.57) | 2.08 (6.82) | 0.51 (3.31) | 1.63 (6.87) | 1.93 (6.63) | 3.08 (8.74) |
| School Counselling services | 0.12 (1.43) | 0.39 (2.23) | 0.64 (3.45) | 0.28 (2.17) | 0.83 (3.98) | 0.46 (2.17) | 1.15 (4.79) |

*Note.* Percentage of participants is displayed by study groups are provided for the categories of the physical health and mental health outcomes. Means and standard deviations (between brackets) are displayed for the remaining outcomes.

CG = Control group. MDD = Depression group. MDD+EXT = Depression with comorbid externalising disorder group. ANX = Anxiety group. ANX+EXT = Anxiety with comorbid externalising disorder group. ANX+MDD = Comorbid depression and anxiety group. ANX+MDD+EXT = Comorbid depression and anxiety with comorbid externalising disorder group.

*Table S2.* Attrition analysis statistics on the study sample.

|  | Contrast test | Effect size |
| --- | --- | --- |
| Sex | 2.34 | 0.04 |
| Study group^1^ | 553.49 | 0.07 |
| Age | 8.31 | 0.07 |
| Race | 0.91 | 0.02 |
| Physical diseases | 21.75 | 0.02 |
| Parents' education | 0.42 | 0.02 |
| Household income | 3.32 | 0.01 |
| Urbanicity | 2.23 | 0.04 |
| Biological parents living with adolescent | 8.93 | 0.07 |

*Note.* These statistics reflect the results of comparing the whole clinical sample (generalised linear model sample; *N* = 1781) and the sample in symptom network analyses (*n* = 1173).

Contrast tests consisted of *t*-based tests for continuous factors and χ^2^ tests for non-continuous ones. Effect size estimates were Cohen’s *d* (continuous factors) and Cramer’s *V* (for non-continuous factors) statistics.

^1^ Sample size by study group in the symptom network analyses: *n* MDD group = 232, *n* MDD+EXT group = 172, *n* ANX group = 290, *n* ANX+EXT group = 204, *n* ANX+MDD group = 139, *n* ANX+MDD+EXT group = 136.

*Table S3A.* Tetrachoric correlation matrix between symptoms in the major depression disorder group.

|  | ag | d1 | d2 | d3 | d4 | d5 | d6 | d7 | d8 | g1 | p1 | sa1 | so1 | so2 | so3 |
| --- | --- | --- | --- | --- | --- | --- | --- | --- | --- | --- | --- | --- | --- | --- | --- |
| ag | 1 |  |  |  |  |  |  |  |  |  |  |  |  |  |  |
| d1 | -0.02 | 1 |  |  |  |  |  |  |  |  |  |  |  |  |  |
| d2 | -0.15 | 0.76 | 1 |  |  |  |  |  |  |  |  |  |  |  |  |
| d3 | 0.34 | 0.12 | 0.31 | 1 |  |  |  |  |  |  |  |  |  |  |  |
| d4 | 0.05 | -0.02 | -0.08 | 0.12 | 1 |  |  |  |  |  |  |  |  |  |  |
| d5 | -0.01 | -0.02 | 0.33 | 0.01 | -0.07 | 1 |  |  |  |  |  |  |  |  |  |
| d6 | -0.09 | 0.41 | 0.52 | 0.45 | -0.03 | 0.19 | 1 |  |  |  |  |  |  |  |  |
| d7 | 0.24 | 0.28 | 0.13 | 0.27 | 0.07 | 0.21 | 0.68 | 1 |  |  |  |  |  |  |  |
| d8 | 0.2 | 0.06 | 0.2 | 0.17 | -0.02 | 0.13 | 0.36 | 0.36 | 1 |  |  |  |  |  |  |
| g1 | -0.03 | -0.14 | -0.05 | 0.06 | 0.29 | 0.12 | 0.28 | 0.26 | 0.26 | 1 |  |  |  |  |  |
| p1 | 0.28 | 0.2 | 0.09 | 0.13 | 0.03 | -0.28 | -0.01 | 0.08 | 0.12 | -0.33 | 1 |  |  |  |  |
| sa1 | -0.14 | 0.05 | -0.01 | -0.02 | 0.21 | -0.01 | 0.13 | 0.14 | 0.01 | -0.61 | 0.09 | 1 |  |  |  |
| so1 | 0.38 | -0.01 | -0.07 | 0.07 | 0.02 | -0.03 | 0.31 | 0.28 | 0.07 | 0.24 | 0.08 | 0.05 | 1 |  |  |
| so2 | 0.16 | 0.02 | 0.1 | 0.01 | 0.05 | -0.06 | 0.13 | 0.21 | 0.11 | 0.19 | 0.22 | 0.08 | 0.31 | 1 |  |
| so3 | 0.05 | 0.14 | 0.02 | -0.2 | -0.03 | 0.15 | 0.02 | 0.35 | 0.14 | -0.04 | 0.24 | 0 | 0.27 | 0.02 | 1 |

*Note.* The ρ estimate is used.

**Symptoms:** ag = Fearful of being in open space. d1 = Sadness. d2 = Discouraged about things in life most days. d3 = Thought about suicide. d4 = Speak/move more slowly than usual most days. d5 = More trouble concentrating most days. d6 = Low self-esteem. d7 = Felt worse than others most days. d8 = Felt guilty most days. g1 = Felt distress due to worry. p1 = Experience sudden attack. sa1 = Being sad/uncomfortable when apart from attachment person. so1 = Shy/afraid/uncomfortable meeting new people. so2 = Shy/afraid/uncomfortable talking to authority. so3 = Shy/afraid/uncomfortable speaking in class.

*Table S3B.* Tetrachoric correlation matrix between symptoms in the major depression disorder with externalising disorder group.

|  | ag | d1 | d2 | d3 | d4 | d5 | d6 | d7 | d8 | g1 | p1 | sa1 | so1 | so2 | so3 |
| --- | --- | --- | --- | --- | --- | --- | --- | --- | --- | --- | --- | --- | --- | --- | --- |
| ag | 1 |  |  |  |  |  |  |  |  |  |  |  |  |  |  |
| d1 | 0.12 | 1 |  |  |  |  |  |  |  |  |  |  |  |  |  |
| d2 | -0.11 | 0.69 | 1 |  |  |  |  |  |  |  |  |  |  |  |  |
| d3 | 0.29 | 0.27 | 0.39 | 1 |  |  |  |  |  |  |  |  |  |  |  |
| d4 | 0.12 | 0.21 | 0.32 | 0.2 | 1 |  |  |  |  |  |  |  |  |  |  |
| d5 | 0.13 | 0.11 | 0.17 | 0.03 | -0.01 | 1 |  |  |  |  |  |  |  |  |  |
| d6 | -0.09 | 0.35 | 0.47 | 0.45 | 0.43 | 0.14 | 1 |  |  |  |  |  |  |  |  |
| d7 | 0.26 | 0.1 | 0.07 | 0.38 | 0.2 | 0.15 | 0.49 | 1 |  |  |  |  |  |  |  |
| d8 | 0.25 | 0.31 | 0.36 | 0.14 | 0.21 | 0.2 | 0.17 | 0.26 | 1 |  |  |  |  |  |  |
| g1 | -0.28 | -0.07 | 0.14 | 0.08 | 0.28 | -0.03 | 0.29 | 0.19 | 0.35 | 1 |  |  |  |  |  |
| p1 | -0.17 | 0.19 | 0.07 | 0.2 | -0.17 | -0.06 | 0.01 | -0.05 | -0.05 | -0.17 | 1 |  |  |  |  |
| sa1 | 0.36 | -0.22 | 0.03 | -0.07 | 0 | 0.23 | 0.07 | 0.22 | -0.07 | -0.47 | 0 | 1 |  |  |  |
| so1 | 0.18 | -0.08 | -0.05 | 0.17 | 0.08 | -0.08 | 0.24 | 0.26 | -0.03 | 0.36 | 0.1 | 0.04 | 1 |  |  |
| so2 | 0.07 | 0.05 | 0.09 | 0 | 0.02 | -0.1 | 0.28 | 0.02 | -0.09 | 0.38 | 0.05 | 0.23 | 0.3 | 1 |  |
| so3 | -0.08 | 0.02 | -0.32 | -0.11 | -0.02 | 0.02 | 0.15 | -0.07 | 0.11 | 0.08 | 0.24 | -0.17 | 0.44 | 0.06 | 1 |

*Note.* The ρ estimate is used.

**Symptoms:** ag = Fearful of being in open space. d1 = Sadness. d2 = Discouraged about things in life most days. d3 = Thought about suicide. d4 = Speak/move more slowly than usual most days. d5 = More trouble concentrating most days. d6 = Low self-esteem. d7 = Felt worse than others most days. d8 = Felt guilty most days. g1 = Felt distress due to worry. p1 = Experience sudden attack. sa1 = Being sad/uncomfortable when apart from attachment person. so1 = Shy/afraid/uncomfortable meeting new people. so2 = Shy/afraid/uncomfortable talking to authority. so3 = Shy/afraid/uncomfortable speaking in class.

*Table S3C.* Tetrachoric correlation matrix between symptoms in the anxiety disorder group.

|  | ag | d1 | d2 | d3 | d4 | d5 | d6 | d7 | d8 | g1 | p1 | sa1 | so1 | so2 | so3 |
| --- | --- | --- | --- | --- | --- | --- | --- | --- | --- | --- | --- | --- | --- | --- | --- |
| ag | 1 |  |  |  |  |  |  |  |  |  |  |  |  |  |  |
| d1 | -0.17 | 1 |  |  |  |  |  |  |  |  |  |  |  |  |  |
| d2 | -0.28 | 0.74 | 1 |  |  |  |  |  |  |  |  |  |  |  |  |
| d3 | 0.33 | 0.12 | 0.54 | 1 |  |  |  |  |  |  |  |  |  |  |  |
| d4 | -0.09 | 0.24 | 0.43 | 0.25 | 1 |  |  |  |  |  |  |  |  |  |  |
| d5 | -0.06 | -0.11 | 0.05 | 0.06 | -0.03 | 1 |  |  |  |  |  |  |  |  |  |
| d6 | -0.23 | 0.54 | 0.67 | 0.46 | 0.21 | 0.1 | 1 |  |  |  |  |  |  |  |  |
| d7 | 0.2 | 0.38 | 0.33 | 0.34 | 0.11 | 0.04 | 0.62 | 1 |  |  |  |  |  |  |  |
| d8 | 0.16 | 0.2 | 0.2 | 0.21 | 0.14 | 0.17 | 0.38 | 0.11 | 1 |  |  |  |  |  |  |
| g1 | 0.04 | -0.11 | 0.09 | 0.15 | 0.23 | 0.24 | 0.32 | 0.26 | 0.34 | 1 |  |  |  |  |  |
| p1 | -0.26 | 0.2 | 0.16 | 0.04 | 0.11 | 0.07 | 0.08 | -0.08 | 0.02 | -0.32 | 1 |  |  |  |  |
| sa1 | -0.25 | -0.16 | 0.08 | 0.06 | 0.23 | 0.35 | 0.17 | 0.03 | 0.01 | -0.39 | 0.23 | 1 |  |  |  |
| so1 | 0.27 | 0.14 | 0.13 | 0.4 | 0.25 | -0.15 | 0.29 | 0.29 | 0.15 | 0.21 | -0.01 | 0.07 | 1 |  |  |
| so2 | 0.18 | 0.08 | 0.13 | -0.03 | 0.1 | 0.13 | 0.14 | 0.18 | 0.06 | 0.27 | 0.04 | 0.07 | 0.23 | 1 |  |
| so3 | 0.14 | 0.27 | 0.14 | -0.13 | 0.2 | 0.17 | 0.06 | 0.25 | 0.4 | 0.31 | 0.13 | -0.22 | 0.03 | 0.14 | 1 |

*Note.* The ρ estimate is used.

**Symptoms:** ag = Fearful of being in open space. d1 = Sadness. d2 = Discouraged about things in life most days. d3 = Thought about suicide. d4 = Speak/move more slowly than usual most days. d5 = More trouble concentrating most days. d6 = Low self-esteem. d7 = Felt worse than others most days. d8 = Felt guilty most days. g1 = Felt distress due to worry. p1 = Experience sudden attack. sa1 = Being sad/uncomfortable when apart from attachment.

*Table S3D.* Tetrachoric correlation matrix between symptoms in the anxiety disorder and comorbid externalising disorder group.

|  | ag | d1 | d2 | d3 | d4 | d5 | d6 | d7 | d8 | g1 | p1 | sa1 | so1 | so2 | so3 |
| --- | --- | --- | --- | --- | --- | --- | --- | --- | --- | --- | --- | --- | --- | --- | --- |
| ag | 1 |  |  |  |  |  |  |  |  |  |  |  |  |  |  |
| d1 | -0.21 | 1 |  |  |  |  |  |  |  |  |  |  |  |  |  |
| d2 | -0.43 | 0.8 | 1 |  |  |  |  |  |  |  |  |  |  |  |  |
| d3 | 0.15 | 0.54 | 0.41 | 1 |  |  |  |  |  |  |  |  |  |  |  |
| d4 | -0.1 | 0.34 | 0.22 | 0.29 | 1 |  |  |  |  |  |  |  |  |  |  |
| d5 | -0.01 | -0.11 | 0.04 | 0.02 | 0.03 | 1 |  |  |  |  |  |  |  |  |  |
| d6 | -0.33 | 0.44 | 0.42 | 0.58 | 0.26 | 0.05 | 1 |  |  |  |  |  |  |  |  |
| d7 | 0.2 | 0.34 | 0.2 | 0.36 | 0.26 | 0.03 | 0.38 | 1 |  |  |  |  |  |  |  |
| d8 | 0.16 | 0.42 | 0.33 | 0.38 | 0.04 | 0.15 | 0.42 | 0.35 | 1 |  |  |  |  |  |  |
| g1 | -0.09 | -0.24 | -0.18 | 0.03 | 0.17 | 0.17 | 0.35 | 0.41 | -0.01 | 1 |  |  |  |  |  |
| p1 | -0.04 | 0.33 | 0.24 | 0.14 | -0.01 | -0.3 | -0.09 | 0.06 | 0.12 | -0.04 | 1 |  |  |  |  |
| sa1 | 0.25 | 0.05 | -0.12 | -0.03 | 0.11 | -0.01 | 0.06 | 0.14 | 0 | -0.27 | -0.05 | 1 |  |  |  |
| so1 | 0.18 | -0.16 | -0.1 | 0.21 | -0.02 | -0.03 | 0.21 | 0.21 | 0.04 | 0.35 | -0.23 | 0.22 | 1 |  |  |
| so2 | 0.24 | -0.08 | -0.1 | 0.12 | 0.02 | -0.02 | 0.09 | -0.05 | -0.04 | 0.45 | 0.05 | 0.04 | 0.09 | 1 |  |
| so3 | 0.06 | 0.17 | 0.05 | -0.01 | 0.14 | 0.17 | 0.16 | 0.38 | 0.2 | 0.18 | 0.08 | 0.12 | 0.24 | -0.03 | 1 |

*Note.* The ρ estimate is used.

**Symptoms:** ag = Fearful of being in open space. d1 = Sadness. d2 = Discouraged about things in life most days. d3 = Thought about suicide. d4 = Speak/move more slowly than usual most days. d5 = More trouble concentrating most days. d6 = Low self-esteem. d7 = Felt worse than others most days. d8 = Felt guilty most days. g1 = Felt distress due to worry. p1 = Experience sudden attack. sa1 = Being sad/uncomfortable when apart from attachment.

*Table S3E.* Tetrachoric correlation matrix between symptoms in the internalising disorder group.

|  | ag | d1 | d2 | d3 | d4 | d5 | d6 | d7 | d8 | g1 | p1 | sa1 | so1 | so2 | so3 |
| --- | --- | --- | --- | --- | --- | --- | --- | --- | --- | --- | --- | --- | --- | --- | --- |
| ag | 1 |  |  |  |  |  |  |  |  |  |  |  |  |  |  |
| d1 | -0.12 | 1 |  |  |  |  |  |  |  |  |  |  |  |  |  |
| d2 | -0.26 | -0.01 | 1 |  |  |  |  |  |  |  |  |  |  |  |  |
| d3 | 0.07 | 0.07 | 0.5 | 1 |  |  |  |  |  |  |  |  |  |  |  |
| d4 | 0.42 | -0.31 | 0.14 | 0.21 | 1 |  |  |  |  |  |  |  |  |  |  |
| d5 | -0.03 | -0.1 | -0.11 | 0.19 | 0.49 | 1 |  |  |  |  |  |  |  |  |  |
| d6 | -0.12 | 0.65 | 0.28 | 0.45 | 0.04 | 0.07 | 1 |  |  |  |  |  |  |  |  |
| d7 | 0.26 | 0.58 | 0.2 | 0.45 | -0.01 | 0.2 | 0.67 | 1 |  |  |  |  |  |  |  |
| d8 | 0.11 | 0.15 | 0.6 | 0.29 | 0.31 | 0 | 0.46 | 0.48 | 1 |  |  |  |  |  |  |
| g1 | -0.48 | 0.29 | 0.48 | 0.22 | 0.1 | 0.34 | 0.57 | 0.49 | 0.38 | 1 |  |  |  |  |  |
| p1 | -0.16 | -0.13 | 0.27 | 0.05 | 0.17 | -0.07 | -0.19 | -0.38 | -0.12 | -0.04 | 1 |  |  |  |  |
| sa1 | 0.19 | -0.13 | -0.3 | -0.06 | 0.29 | -0.07 | 0.25 | -0.12 | -0.2 | 0.07 | -0.06 | 1 |  |  |  |
| so1 | 0.3 | -0.11 | 0.13 | 0.07 | -0.15 | -0.42 | 0.05 | 0.35 | 0.06 | 0.18 | 0 | 0.27 | 1 |  |  |
| so2 | 0.07 | -0.3 | -0.09 | -0.31 | 0.01 | 0.12 | 0.03 | 0.09 | 0.26 | 0.1 | 0.05 | 0.04 | 0.28 | 1 |  |
| so3 | 0.41 | -0.16 | 0.1 | -0.09 | 0.24 | 0.25 | -0.24 | 0.16 | 0.25 | -0.07 | 0.09 | -0.12 | 0.13 | 0.29 | 1 |

*Note.* The ρ estimate is used.

**Symptoms:** ag = Fearful of being in open space. d1 = Sadness. d2 = Discouraged about things in life most days. d3 = Thought about suicide. d4 = Speak/move more slowly than usual most days. d5 = More trouble concentrating most days. d6 = Low self-esteem. d7 = Felt worse than others most days. d8 = Felt guilty most days. g1 = Felt distress due to worry. p1 = Experience sudden attack. sa1 = Being sad/uncomfortable when apart from attachment.

*Table S3F.* Tetrachoric correlation matrix between symptoms in the internalising disorder with comorbid externalising disorder group.

|  | ag | d1 | d2 | d3 | d4 | d5 | d6 | d7 | d8 | g1 | p1 | sa1 | so1 | so2 | so3 |
| --- | --- | --- | --- | --- | --- | --- | --- | --- | --- | --- | --- | --- | --- | --- | --- |
| ag | 1 |  |  |  |  |  |  |  |  |  |  |  |  |  |  |
| d1 | 0.11 | 1 |  |  |  |  |  |  |  |  |  |  |  |  |  |
| d2 | -0.12 | 0.75 | 1 |  |  |  |  |  |  |  |  |  |  |  |  |
| d3 | 0.07 | 0.44 | 0.38 | 1 |  |  |  |  |  |  |  |  |  |  |  |
| d4 | 0.04 | 0.08 | -0.08 | 0.08 | 1 |  |  |  |  |  |  |  |  |  |  |
| d5 | 0.15 | 0.28 | 0 | -0.14 | 0.28 | 1 |  |  |  |  |  |  |  |  |  |
| d6 | -0.21 | 0.51 | 0.44 | 0.35 | 0.02 | 0.17 | 1 |  |  |  |  |  |  |  |  |
| d7 | -0.02 | 0.49 | 0.43 | 0.36 | -0.12 | 0.16 | 0.6 | 1 |  |  |  |  |  |  |  |
| d8 | 0.44 | 0.03 | 0.06 | 0.09 | -0.06 | -0.07 | 0.17 | 0.28 | 1 |  |  |  |  |  |  |
| g1 | 0.37 | 0.54 | 0.33 | 0.39 | 0.17 | 0.1 | 0.46 | 0.23 | 0.45 | 1 |  |  |  |  |  |
| p1 | -0.43 | -0.21 | -0.1 | 0.06 | 0.04 | -0.18 | 0.07 | -0.07 | -0.05 | -0.18 | 1 |  |  |  |  |
| sa1 | 0.14 | -0.26 | -0.16 | -0.06 | 0.25 | 0.13 | 0.29 | -0.05 | 0.19 | -0.07 | 0.01 | 1 |  |  |  |
| so1 | 0.2 | -0.09 | -0.13 | 0.06 | 0.09 | 0.15 | 0.12 | -0.08 | -0.06 | 0.51 | -0.09 | 0.12 | 1 |  |  |
| so2 | 0.33 | 0.03 | -0.04 | 0.05 | -0.2 | -0.12 | 0.31 | 0.07 | 0.19 | 0.5 | 0.04 | 0.15 | 0.3 | 1 |  |
| so3 | -0.05 | 0.4 | -0.02 | -0.1 | -0.04 | 0.14 | 0.29 | 0.27 | 0.13 | 0.48 | 0.19 | -0.2 | 0.23 | 0.22 | 1 |

*Note.* The ρ estimate is used.

**Symptoms:** ag = Fearful of being in open space. d1 = Sadness. d2 = Discouraged about things in life most days. d3 = Thought about suicide. d4 = Speak/move more slowly than usual most days. d5 = More trouble concentrating most days. d6 = Low self-esteem. d7 = Felt worse than others most days. d8 = Felt guilty most days. g1 = Felt distress due to worry. p1 = Experience sudden attack. sa1 = Being sad/uncomfortable when apart from attachment.

*Table S4.* Clustering measures for the estimated networks.

| Study group | Transitivity | Average of shortest paths | Small worldness |
| --- | --- | --- | --- |
| MDD | 0.35 | 1.97 | 1.08 |
| MDD+EXT | 0.33 | 1.71 | 0.94 |
| ANX | 0.51 | 1.59 | 1.02 |
| ANX+EXT | 0.55 | 2.04 | 1.24 |
| ANX+MDD | 0.30 | 1.93 | 0.97 |
| ANX+MDD+EXT | 0.60 | 1.77 | 1.26 |

*Note.* MDD = Depression group. MDD+EXT = Depression with comorbid externalising disorder group. ANX = Anxiety group. ANX+EXT = Anxiety with comorbid externalising disorder group. ANX+MDD = Comorbid depression and anxiety group. ANX+MDD+EXT = Comorbid depression and anxiety with comorbid externalising disorder group.

## *Figure S1.* Density plot of observed and imputed data.

*
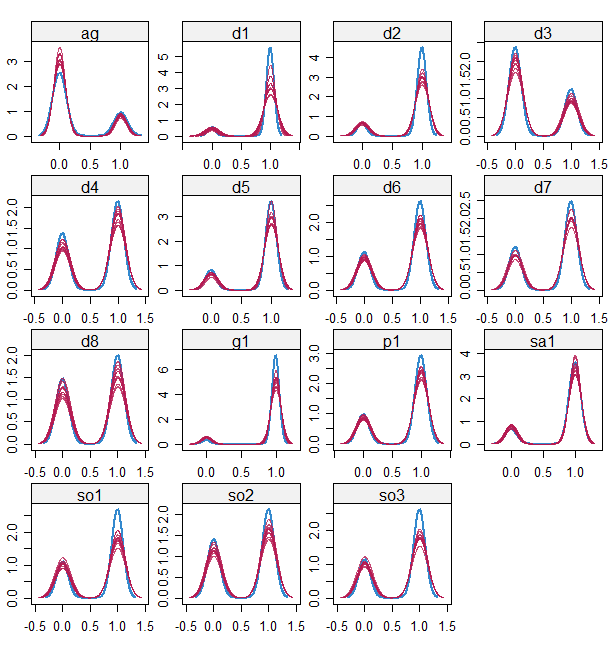
*

*Note.* This figure displays the cumulative density distribution of observed data, in blue ink; and those from imputed data (all iterations), in red ink.

**Symptoms:** ag = Fearful of being in open space. d1 = Sadness. d2 = Discouraged about things in life most days. d3 = Thought about suicide. d4 = Talk/move more slowly than usual most days. d5 = More trouble concentrating most days. d6 = Low self-esteem. d7 = Felt not as good as others most days. d8 = Felt guilty most days. g1 = Felt distress due to worry. p1 = Experience sudden attack. sa1 = Being sad/uncomfortable when apart from attachment person. so1 = Shy/afraid/uncomfortable meeting new people. so2 = Shy/afraid/uncomfortable talking to authority. so3 = Shy/afraid/uncomfortable speaking in class.

## *Figure S2.* Bootstrapped confidence intervals of estimated edge-weights for the estimated networks.

## ***
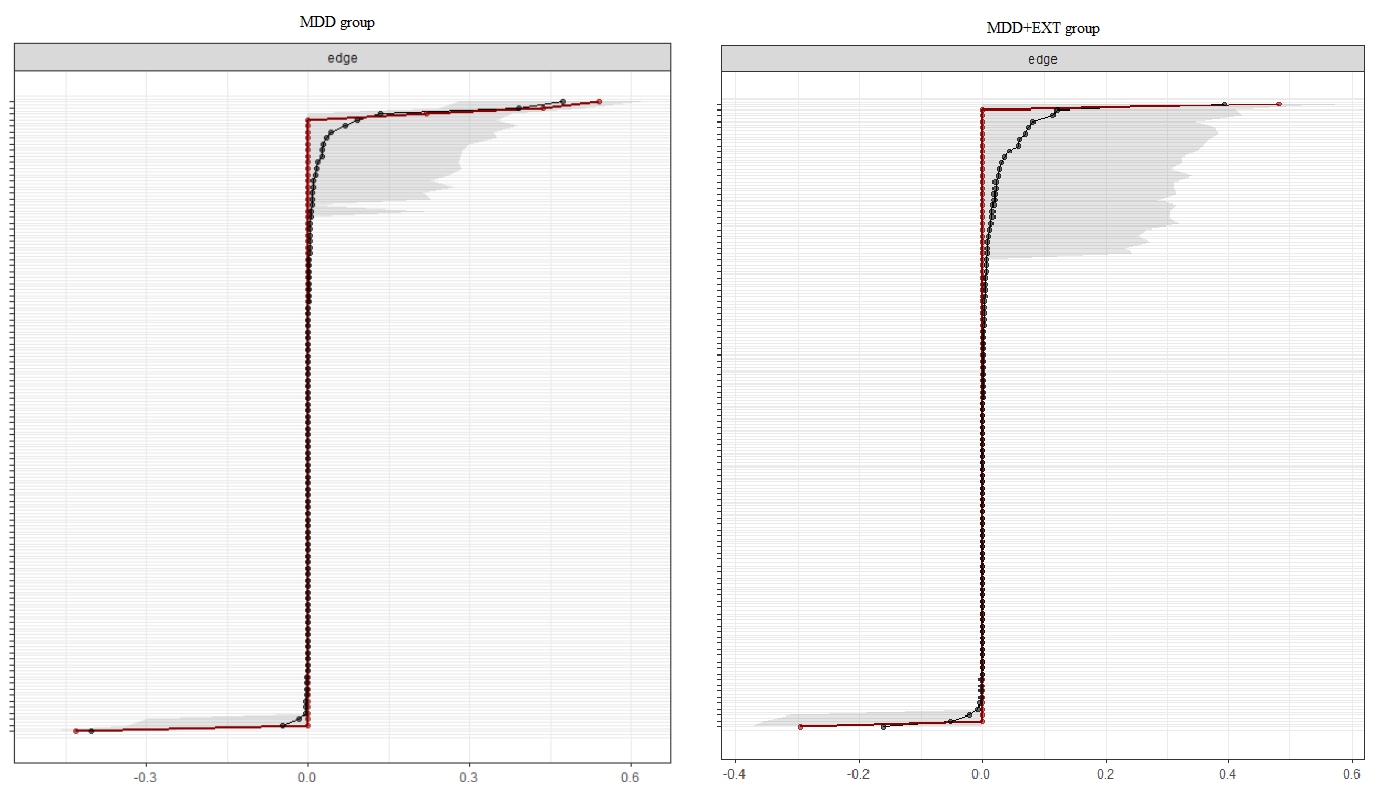
***

## ***
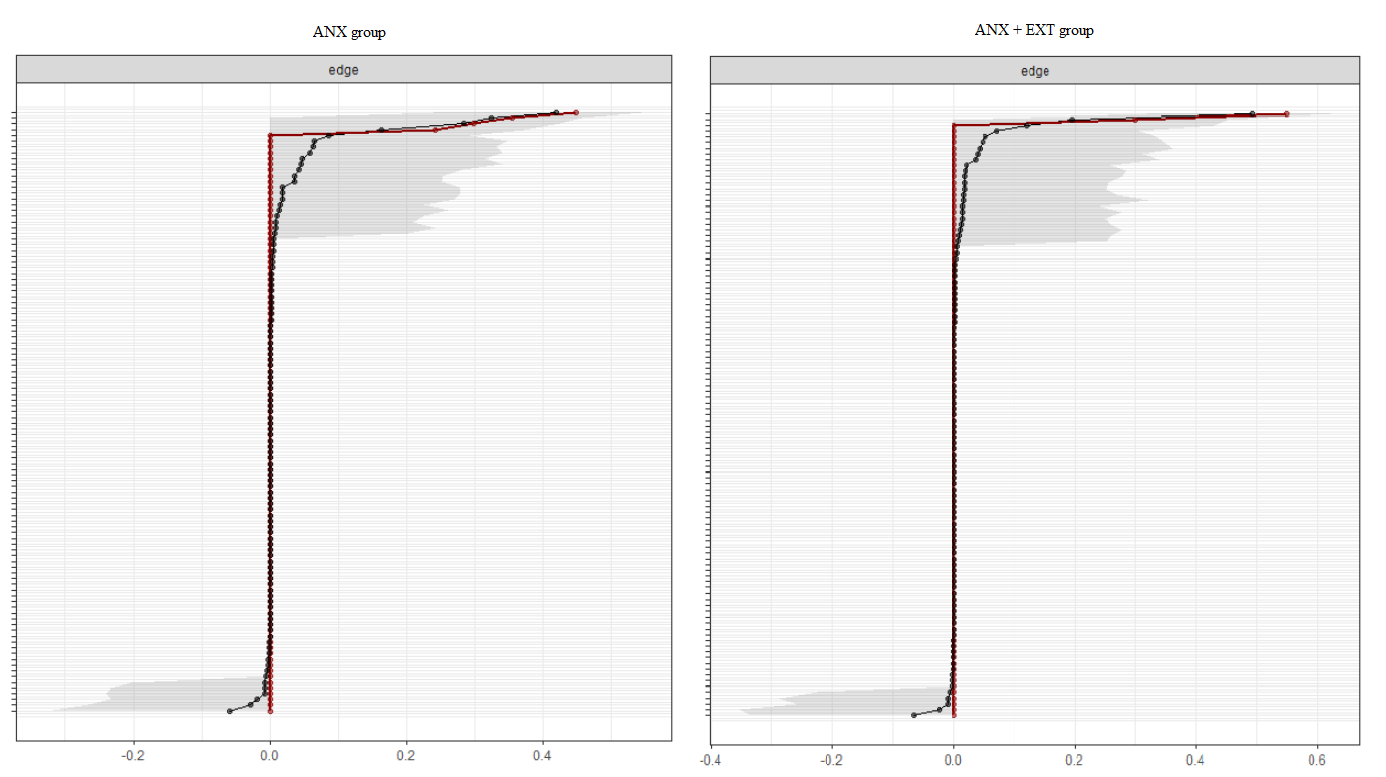
***

## ***
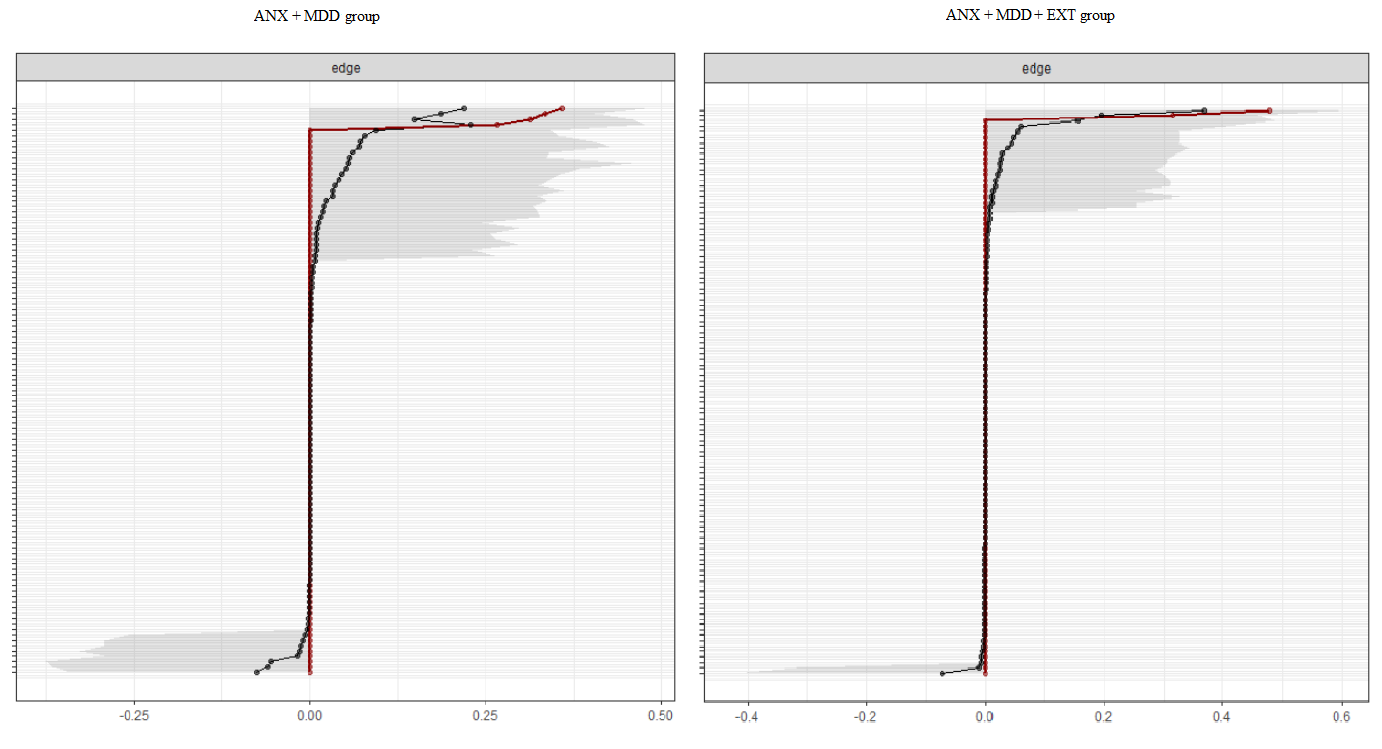
***

## ***Note.*** The red line represents the sample values, the black line the mean of bootstrapped samples and the shaded area the bootstrapped 95% confidence interval. All the items are displayed, ordered from the edge with the highest edge-weight to the edge with the lowest edge-weight.
